# Supplementary material for: Challenges in global climate models to represent cloud response to aerosols: insights from volcanic eruptions
Source: Nat Commun. 2025 Dec 18;17:627. doi: 10.1038/s41467-025-67359-3 (PMC12816603; doi:10.1038/s41467-025-67359-3)
Supplement: Supplementary file 1 — Supplementary Information [file 41467_2025_67359_MOESM1_ESM.pdf]

Supplementary Information for

"Challenges in global climate models to represent cloud response to aerosol: insights from volcanic eruptions"

Yu Wang[1*], David Neubauer[2], Ying Chen[3], George Jordan[4], Florent Malavelle[5], Tianle Yuan[6,7], Daniel Partridge[8], Paul Field[5,9], Hao Wang[10], Minghuai Wang[10], Martine Michou[11], Pierre Nabat[11], Anton Laakso[12], Gunnar Myhre[13], & Ulrike Lohmann[2]

[1]School of GeoSciences, University of Edinburgh, Edinburgh, UK

[2]Institute for Atmospheric and Climate Science, ETH Zürich, Zürich, Switzerland

[3]School of Geography, Earth and Environmental Sciences, University of Birmingham, Birmingham, UK

[4]Met Office Hadley Centre, Exeter, UK

[5]Met Office, Exeter, UK

[6]Goddard Earth Sciences Technology and Research (GESTAR) II, University of Maryland, Baltimore County, Baltimore, MD, USA.

[7]Sciences and Exploration Directorate, Goddard Space Flight Center, Greenbelt, MD, USA.

[8]College of Engineering, Mathematics, and Physical Sciences, University of Exeter, Exeter, UK

[9]School of Earth and Environment, University of Leeds, Leeds, UK

[10]School of Atmospheric Sciences, Nanjing University, Nanjing, China

[11]Météo-France, CNRS, Univ. Toulouse, CNRM, Toulouse, France

[12]Finnish Meteorological Institute, Kuopio, Finland

[13]CICERO Center for International Climate Research Oslo, Norway

*Corresponding author: Yu Wang (y.w@ed.ac.uk)

Table-of-Contents:

Supplementary Discussion Section S1

Supplementary Figures S1-S3

Supplementary Discussion Section S1.
Spatial distribution of cloud property changes in observations and models

The spatial distributions of key cloud properties and the influence of Holuhraun-2014 volcanic plumes are shown in Supplementary Figures S1-S3, with observations[1] provided in the top row and simulations from six models in the following rows.

Supplementary Fig. S1 shows the baseline of the control case, i.e. without volcanic eruption. A large divergence is found among six models. For example: regarding cloud droplet number concentration (Nd), CESM2.1.0 underestimates by about 70%, but CNRM-ESM2-1 overestimates by about 40%; regarding droplet effective radius (Re), ECHAM6.3-SALSA2.0 underestimates by about 65%, and other models slightly underestimate; regarding liquid water path (LWP), UKESM1 and CNRM-ESM2-1 underestimate by 50-70% and other models slightly underestimate; regarding liquid cloud cover (LCC), UKESM1 and CNRM-ESM2-1 provide a reasonable estimate, ECHAM6.3-HAM2.3, ECHAM6.3-SALSA2.0 and CESM2.1.0 underestimate by 35-65%, while CAM5.3_Oslo overestimates by about 50%. Supplementary Fig. S2 shows the volcanic case, in which both large overestimation and underestimation of all cloud variables are observed. This large variation of baseline and experimental simulations is expected and is in line with previous studies, showing great diversity of the chosen models and also justifying the importance of looking at the relative change of clouds caused by aerosol (rather than absolute change)[2], which is the cloud susceptibility in a logarithmic scale that we discuss in the main text.

Supplementary Fig. S3 provides the difference between the volcanic case and baseline, i.e. Supplementary Fig. S2 minus Supplementary Fig. S1, which most intuitively shows the region influenced by the volcanic plume. Generally speaking, over the whole North Atlantic, observation shows a clear increase of Nd, a decrease of Re, no clear change of LWP (only -0.03 g m^{-2} for domain-average), and a large increase of LCC; models well capture the spatial patterns of ΔNd and ΔRe (Twomey effect). Four out of six models largely overestimate the increase of LWP, with only UKESM1 and CNRM-ESM2-1 showing a reasonable regional response of LWP, in line with Malavelle et al.[3]. However, all models show negligible increases in LCC and fail to reproduce the observed strong increase in cloud cover.

These model-observation intercomparison highlights the model’s incapability to reproduce the key cloud properties and aerosol-induced cloud responses as observed, especially the LWP and LCC. Although Re and LWP are determined mainly by cloud microphysics diagnostically, while LCC is diagnosed by grid-mean relative humidity, these key cloud variables are interlinked through the partitioning of water in the vapour, liquid, and ice phases in models. To further understand model bias and its capability to represent the observation, we applied different cloud microphysics and cloud cover schemes and conducted sensitivity studies of cloud microphysical processes, see detailed discussion in the main text.

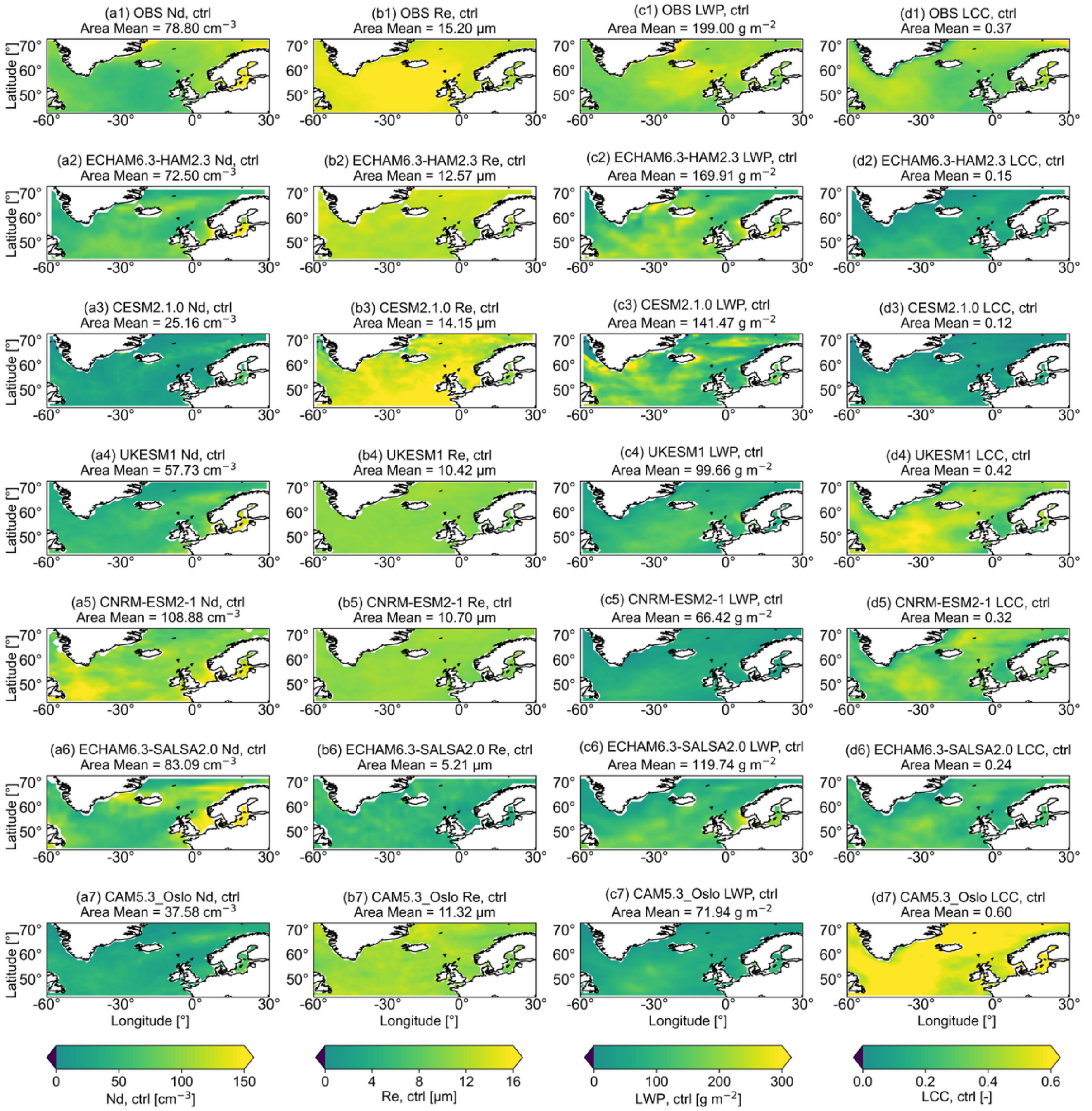

Supplementary Fig. S1. The **spatial distribution** of cloud droplet number concentration (Nd), effective radius (Re), liquid water path (LWP), and liquid cloud cover (LCC) in Oct. 2014 (corresponding to columns from left to right), **for the control case**, i.e. without volcanic eruption. Rows from top to bottom are: (**a1-d1**) observations derived from a combination of satellite and machine learning[1]; (**a2-d2**) ECHAM6.3-HAM2.3; (**a3-d3**) CESM2.1.0; (**a4-d4**) UKESM1; (**a5-d5**) CNRM-ESM2-1; (**a6-d6**) ECHAM6.3-SALSA2.0; (**a7-d7**) CAM5.3_Oslo.

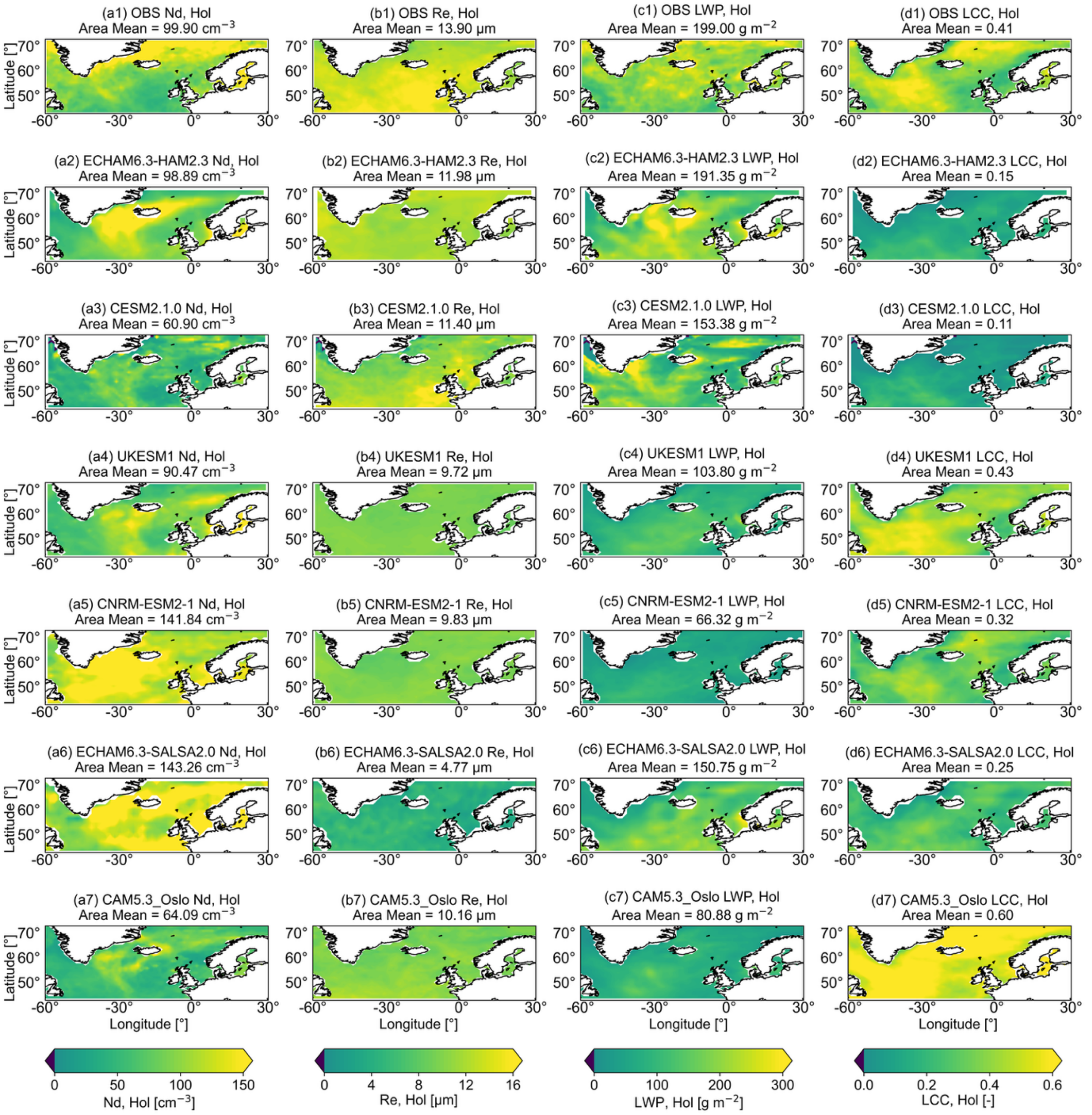

Supplementary Fig. S2. The **spatial distribution** of cloud droplet number concentration (Nd), effective radius (Re), liquid water path (LWP), and liquid cloud cover (LCC) in Oct. 2014 (corresponding to columns from left to right), **for the volcano case**. Rows from top to bottom are: (**a1-d1**) satellite observations[1]; (**a2-d2**) ECHAM6.3-HAM2.3; (**a3-d3**) CESM2.1.0; (**a4-d4**) UKESM1; (**a5-d5**) CNRM-ESM2-1; (**a6-d6**) ECHAM6.3-SALSA2.0; (**a7-d7**) CAM5.3_Oslo.

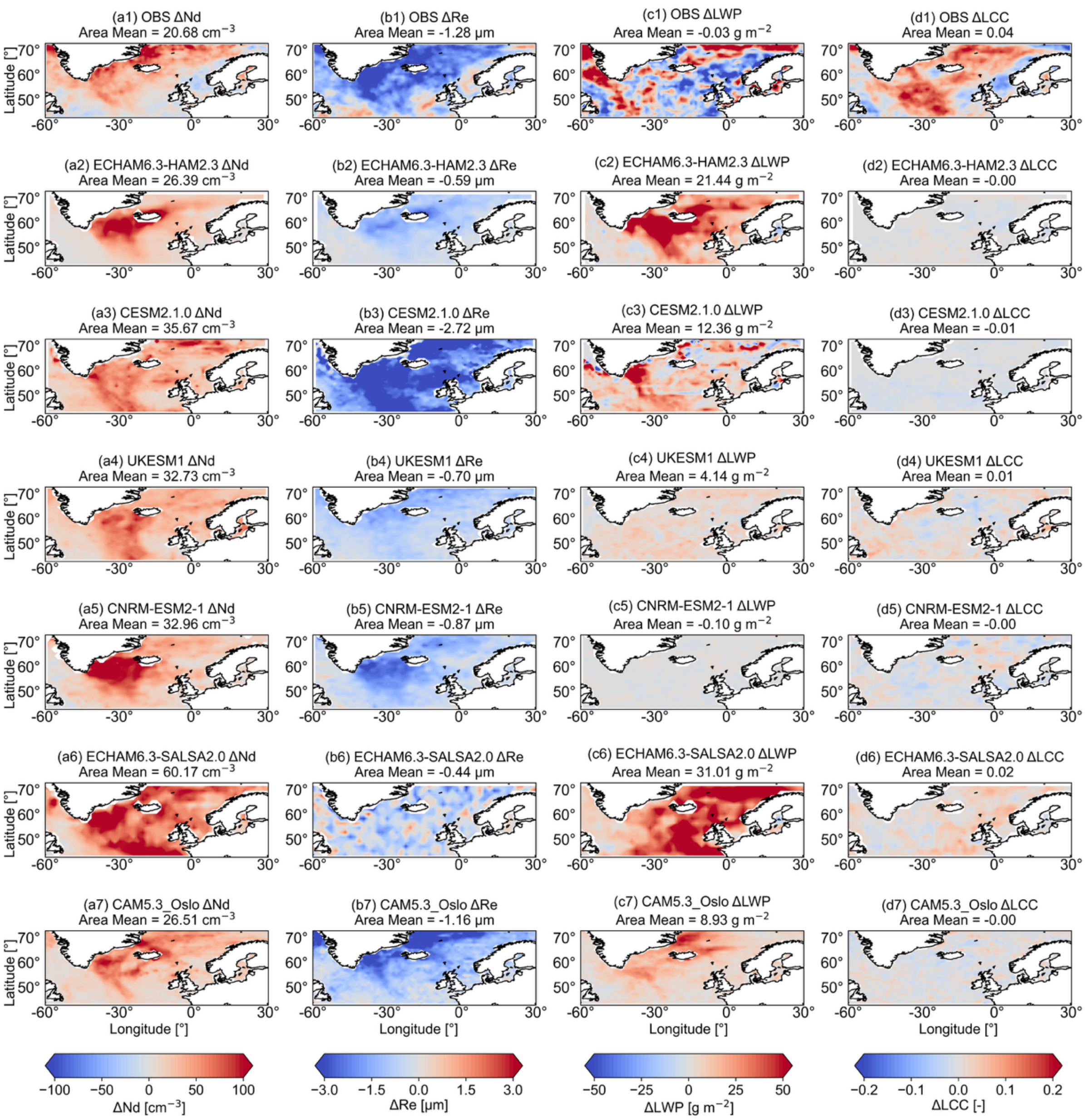

Supplementary Fig. S3. The **spatial distribution** of cloud droplet number concentration (Nd), effective radius (Re), liquid water path (LWP), and liquid cloud cover (LCC) in Oct. 2014 (corresponding to columns from left to right), for the **differences between the volcano and control cases**. Rows from top to bottom are: (**a1-d1**) satellite observations minus machine learning derived observations of control case[1]; (**a2-d2**) ECHAM6.3-HAM2.3; (**a3-d3**) CESM2.1.0; (**a4-d4**) UKESM1; (**a5-d5**) CNRM-ESM2-1; (**a6-d6**) ECHAM6.3-SALSA2.0; (**a7-d7**) CAM5.3_Oslo.

References for Supplementary Information:

1. Chen Y*, et al.* Machine learning reveals climate forcing from aerosols is dominated by increased cloud cover. *Nature Geoscience* **15**, 609-614 (2022).

2. Ghan S*, et al.* Challenges in constraining anthropogenic aerosol effects on cloud radiative forcing using present-day spatiotemporal variability. *Proceedings of the National Academy of Sciences* **113**, 5804-5811 (2016).

3. Malavelle FF*, et al.* Strong constraints on aerosol–cloud interactions from volcanic eruptions. *Nature* **546**, 485-491 (2017).
